# Synthesis, physico-mechanical and microstructural characterization of Al6063/SiC/PKSA hybrid reinforced composites

**DOI:** 10.1038/s41598-021-94420-0

**Published:** 2021-07-21

**Authors:** P. P. Ikubanni, M. Oki, A. A. Adeleke, P. O. Omoniyi

**Affiliations:** 1 Department of Mechanical Engineering, College of Engineering, Landmark University, Omu-Aran, Kwara State Nigeria; 2 Department of Mechanical Engineering Science, University of Johannesburg, Johannesburg, South Africa

**Keywords:** Mechanical engineering, Materials science

## Abstract

The utilization of agro-residues ash as complementary reinforcing materials continues to gain prominence for metal matrix composite (MMCs) development. A rarely investigated but largely available ash among these agro-residues is the palm kernel shell ash (PKSA). Thus, the present study investigates the influence of PKSA particulates hybridized with SiC on the physico-mechanical properties and microstructure of Al6063 metal composites. The composites are synthesized using the double stir-casting technique with SiC held constant at 2 wt.%, while the PKSA contents are varied from 0 to 8 wt.%. The phases present and morphology of the composites are investigated using X-ray diffractometer (XRD) and scanning electron microscopy (SEM), respectively. The density, porosity, hardness, tensile and fracture toughness tests are carried out on the hybrid composites. X-ray diffractometer revealed that for Al 6063, only Al cubic crystal system was identifiable within the matrix. However, for the reinforced composites, major phases identified are Al, Fe_3_Si, SiC, MgO, and SiO_2_. The SEM images show that the particulates reinforcements (SiC and PKSA) were uniformly dispersed in the matrix. The percentage porosity for the composites ranged from 2.06 to 2.39%. In addition, hardness, yield strength and ultimate tensile strength of the composites are about 10.3%, 18.5% and 10.4%, respectively better than for Al 6063. However, the percent elongation and fracture toughness are lower for the hybrid composites than for Al 6063 and SiC reinforced composite with values decreasing with increase in ash content. Hence, the MMCs produced will be applicable for light-weight engineering applications.

## Introduction

The development of new materials for industrial technology with various applications is growing fast^1–3^. The search for new materials has been attributed to the limitations of conventional metals and alloys for the attainment of good strength combination, resistance to wear, toughness, high temperature performance, as well as resistance to corrosion^2,4–6^. Aluminium matrix composites (AMCs) are new materials that has become a major attraction to researchers for some decades. AMCs have been widely utilized in the automobile, aerospace, sport, manufacturing industries and many more^1,7–10^. However, there has been continuous efforts to improve the existing AMCs as well as developing novel types for various applications.

The utilization of single or hybrid ceramic reinforcements in AMCS has been the focus of some studies^6,11,12^. For the suitability of usage of AMC produced using Al matrix and SiC as reinforcement, Poornesh et al.^12^ studied the mechanical properties of AMCs produced through stir casting technique using Al-18wt%Si as matrix and SiC as reinforcement. The hardness and tensile strength of the AMC produced were better compared to the unreinforced Al-alloy, though it was the opposite for the toughness of the samples. Hybrid ceramic reinforcements (SiC and B_4_C) and AA6082-T6 alloy were used for the development of AMCs by Singh & Goyal^6^. Conventional stir casting method was used with varying weight percentages of reinforcement between 5 and 20 wt.% for the production. It was reported that increase in the reinforcement led to an increase in the hardness and tensile strength with decreasing percentage elongation. The mechanical properties of the hybrid AMCs in the study was better compared with the unreinforced alloy. Rajesh and Kaleemulla^10^ also investigated the mechanical behaviour of AMCs produced using Al7075 alloy reinforced with SiC and Al_2_O_3_ via stir casting method. The mechanical properties of the reinforced Al7075 were better compared to the unreinforced alloy.

Due to the high cost of synthetic ceramic reinforcements, researchers continue to study alternative materials (agro-residues and industrial wastes) which could serve as partial replacement for ceramic reinforcement in AMCs in recent years^3,14–16^. Hence, attention to low cost reinforcing material is growing rapidly. Dinaharan et al.^16^ used rice husk ash (RHA) as the reinforcing materials in AA6061 aluminium matrix for the production of AMC via friction stir processing. The reinforced alloy showed homogenous distribution of RHA particles. The tensile strength of the AMC improved while the fractured surface examined confirmed an excellent interfacial bonding of the RHA particles with the Al matrix. The mechanical and fracture properties as well as microstructure of hybrid AMC reinforced with SiC and groundnut shell ash (GSA) was investigated by Alaneme et al.^14^. The AMCs was produced using two-step stir casting technique. The reinforcement particulates were uniformly distributed in the AMC through the microstructure assessment and the mechanical properties improved.

Several other agro-residues that have been used include bean pod ash^7^ (Aigbodion, 2019), corn cob ash^17^, bagasse ash^15^ and palm kernel shell^18^. Palm kernel shell (PKS) and palm kernel shell ash (PKSA) is also an agro-waste and their usage as reinforcement particulates in AMCs production are limited in literature. PKS and PKSA have been used extensively in reinforcement in concrete^19–21^, biomass briquette and particleboard^22,23^, geopolymer composite^24^, and activated carbon for supercapacitor electrode^25^. PKS is an agro-residue that is abundant in several countries such as Nigeria, Brazil and Malaysia^26–28^. PKSA is obtained from the complete burning of PKS into ash^26^. To reduce environmental pollution, PKS and PKSA could be used as partial reinforcement in the production of metal matrix composites. During the synthesis of a novel surface AMC composite, PKSA reinforcement was included in aluminium substrate surface via the friction stir processing route^13^. More so, a new stir casting method (double layer feeding) was employed in the synthesis of AMCs. The AMCs was developed using 1–4 wt.% PKSA nanoparticles (PKSAnp) in A356 alloy matrix. Uniform distribution of the PKSAnp in the matrix was seen from the morphological analysis, while the mechanical properties of the developed AMCs improved compared to the unreinforced alloy^8^. The influence of PKSA on the mechanical properties of recycled Al alloy obtained from automobile engine block cylinder was investigated by Oladele & Okoro^29^. The results revealed that the PKSA is suitable for usage in some automobile parts production. The study of Edoziuno et al.^18^ evaluated the physical and chemical properties as well as the microstructural characteristics of AMCs produced using novel PKS particulates. The weight percentage range of the PKS was between 2.5 and 15 wt.%. Stir casting technology was employed in the production of the AMCs. The results showed improved physical properties and homogenous distribution of the PKS in the matrix.

However, little or no work has been done on the influence of hybrid reinforcement particulates of PKSA and SiC on the physical and mechanical properties of AMCs. For any developed material to be useful for engineering purposes, the developed composite material should be subjected to various tests to ascertain its properties for real-life applications. Hence, the rationale behind this study is to investigate the influence of hybrid reinforcements of PKSA and SiC on the properties of Al6063 matrix alloy for engineering purposes. Therefore, this study synthesized the AMC using Al-6063 matrix and hybrid reinforcements of SiC and PKSA. This research examines the influence of PKSA produced at 900 $^\circ{\rm C}$ as reinforcement in AMCs. More so, the effects of the particulate composition variation by weight fraction of PKSA and SiC on the physico-mechanical properties as well as on the microstructure of the AMCs produced were investigated. The findings from this study would add to the existing database of cost-effective hybrid reinforcement in aluminium matrix composites.

## Materials and method

### Matrix alloy and reinforcement materials

This study utilized Al6063 as the matrix material. Table 1 displays the elemental compositions of the Al6063 matrix material. To obtain one of the reinforcement materials, PKS was purchased from a local market in Osogbo, Osun State, Nigeria. PKSA and SiC were the reinforcing materials used in the formation of the hybrid AMC. The PKSA was obtained through complete combustion of PKS in a muffle furnace at 900 °C. PKSA obtained from the burning of PKS was sieved to an average size of < 40 $\mathrm{\mu m}$, while the average size of the SiC particulate was about 30 $\mathrm{\mu m}$. Table 1 also displays the chemical oxides present in the produced PKSA.

**Table 1:** Elemental composition of the Al6063 matrix and chemical oxides present in the PKSA.

Constituents	Si	Fe	Mn	Mg	Cu	Ti	Zn	Cr	Sn	Al	
*%	0.43	0.17	0.04	0.48	0.01	0.02	0.01	0.01	0.01	Bal	

Constituents	Na_2_O	MgO	Al_2_O_3_	SiO_2_	P_2_O_5_	K_2_O	CaO	TiO_2_	Fe_2_O_3_	MnO	LOI
**%	0.17	3.14	6.46	66.90	3.78	5.20	5.52	0.53	5.72	0.08	2.50

*Al6063 matrix elemental compositions; **Chemical oxides in PKSA.

### Production of composites

**Figure 1:**
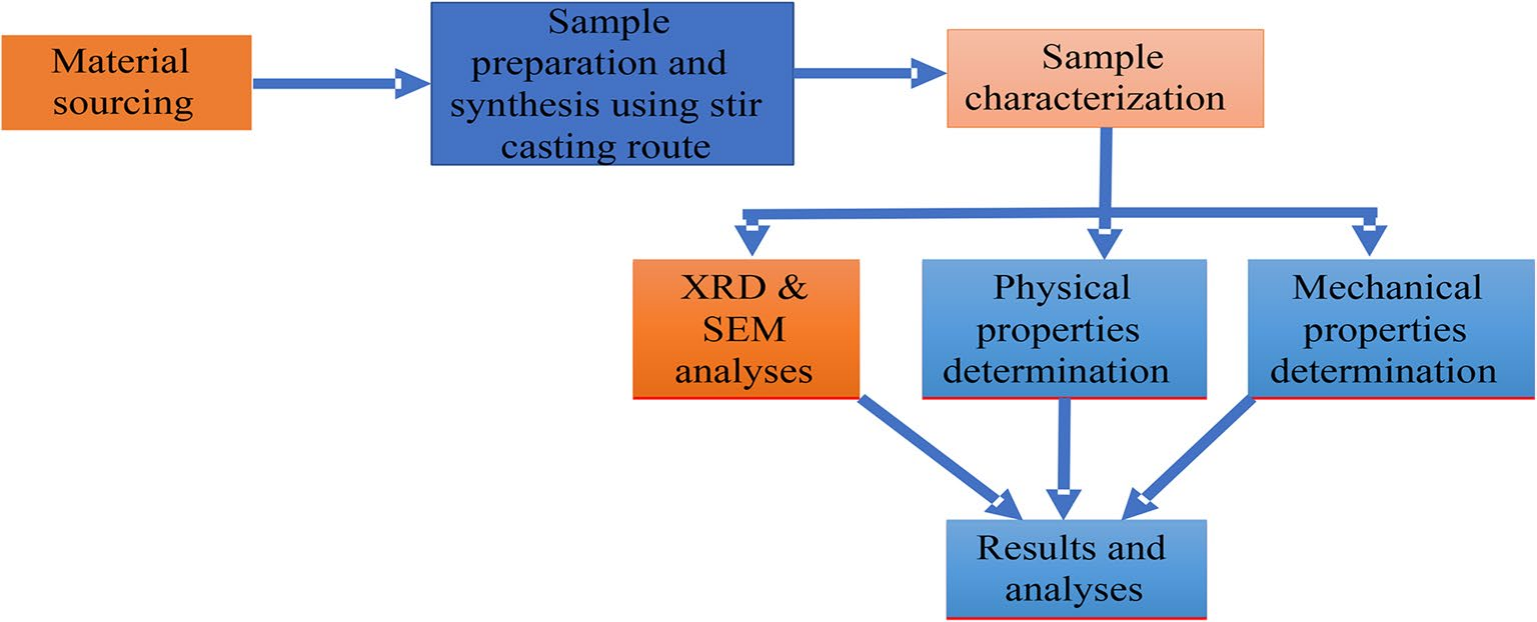
Experimental procedure schematic diagram.

**Figure 2:**
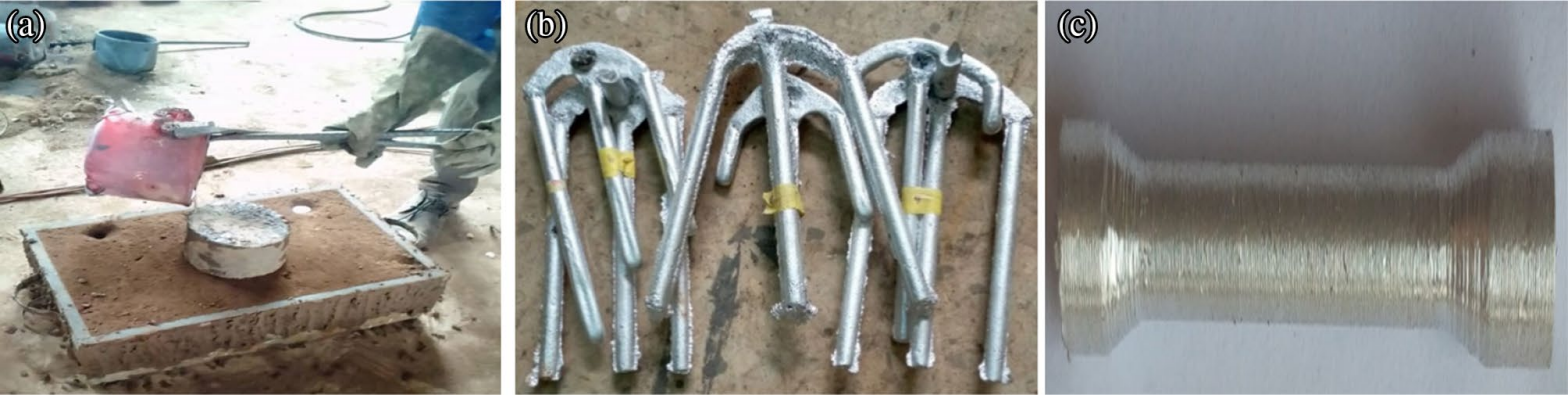
(**a**) Pouring of molten metal composite into prepared sand mold (**b**) solidified metal composite (**c**) tensile test sample.

The technology employed in the production of the composites is the liquid metallurgy route using the two-step stir casting method^14^. The flowchart of the processes involved in this study is shown in Fig. 1. The amount of PKSA and SiC required for the preparation of the 2, 4, 6, 8, and 10 wt.% reinforcements in the matrix was determined through charge calculation consisting of PKSA to SiC ratios (0:0, 0:2, 2:2, 4:2, 6:2, 8:2). To remove moisture inherent in the reinforcements’ particulates and for improved wettability with the Al6063 matrix, they were firstly preheated at 250 °C. The Al6063 matrix ingots were charged into a gas-fired crucible furnace and heated to $750\pm 30^\circ{\rm C}$ (above the alloy’s liquidus temperature) to guarantee the whole matrix alloy melting. The obtained molten matrix alloy was permitted to cool (semi-solid state) in the furnace before charging the preheated reinforcement particulates into it. Thereafter, there was manual stirring of the slurry for 5–10 min before the slurry was superheated to $800\pm 50^\circ{\rm C}$. A mechanical stirrer was utilized for the second stirring of the slurry at a speed of 400 rpm for 10 min. Finally, the melt was poured into sand mould prepared until it solidifies (Fig. 2a,b). The designation of the samples produced is shown in Table 2.

**Table 2:** Composite sample designation.

Sample designation	Composition
A0	Al 6063 alloy
A1	Al 6063 alloy + 2 wt.% SiC
A2	Al 6063 alloy + 2 wt.% PKSA + 2 wt.% SiC
A3	Al 6063 alloy + 4 wt.% PKSA + 2 wt.% SiC
A4	Al 6063 alloy + 6 wt.% PKSA + 2 wt.% SiC
A5	Al 6063 alloy + 8 wt.% PKSA + 2 wt.% SiC

### XRD and microstructural characterization

The X-ray diffractometer utilized to obtain the phases present in the produced composites was PANalytical Empyrean diffractometer, with Cu K_α_ radiation = 1.5406 $\r{A}$, acceleration voltage of 45 kV, and 40 mA current. The scan range (2θ) was between 5 and 90° with 0.026°/min scan speed. The mineral phases were picked using X’Pert Highscore Plus software attached with the diffractometer. A Vega 3 Tescan model SEM with EDS attachment was employed for the microstructural examination of the produced composites. The test specimens were obtained from the cylindrical rods through machine-cut from rods. The samples were metallographically grinded and polished with emery paper of different grits and polishing clothes, then etched with Keller’s reagent. The polished and etched samples were loaded into the SEM machine. The magnification was set (majorly 500 micron) and beam intensity of 14 was used. The Vega software was then used to capture the image from the SEM. The image was scanned unto EDS using Aztec software. The fractured surface morphology of the composites was visualized using the SEM–EDS equipment.

### Physical properties tests

#### Density determination

The principle of Archimedes was used to measure the density of the base metal and the reinforced samples as described by Prasad et al^30^. Each sample was weighed in air (*m*) and distilled water (*m*_*1*_). The mass and other parameters were then used to obtain the density (${\rho }_{c}$) of the composite based on Eq. (1).

1 $${\rho }_{c}=\frac{m}{m-{m}_{1}}{\rho }_{w}$$

where ${\rho }_{c}$, $m$, ${m}_{1}$, and ${\rho }_{w}$ is composite density (kg/m^3^), mass of sample (in air), mass of sample composite in distilled water (kg), and distilled water density (1000 kg/m^3^), respectively.

#### Porosity determination

The method presented by Prasad et al.^30^ was adopted to estimate the porosity of the composite using Eqs. (2)–(3).

2 $$Porosity=\frac{{\rho }_{th}-{\rho }_{m}}{{\rho }_{th}}$$

where ${\rho }_{th}$ and ${\rho }_{m}$ are the theoretical and measured densities (in kg/m^3^), respectively.

Equation (3) is the mathematical relationship of the theoretical density for the hybrid composites. The volume fractions for both reinforcements as well as the matrix were included and calculated using Eq. (3):

3 $${\rho }_{th}={\rho }_{Al}{V}_{Al}+{\rho }_{PKSA}{V}_{PKSA}+{\rho }_{SiC}{V}_{SiC}$$

where ${\rho }_{Al}, {\rho }_{PKSA},{\rho }_{SiC}$ are densities of aluminum matrix, PKSA and SiC, respectively while ${V}_{Al}, {V}_{PKSA},{V}_{SiC}$ are volume fractions of aluminum matrix, PKSA and SiC, respectively.

### Mechanical Tests

#### Hardness test

The hardness test was carried on the samples out based on the ASTM E10-18 standard^31^. Brinell hardness machine was used in the determination of the hardness value. On each specimen, four indents were done and readings were obtained for reliability of the data. The average hardness value was computed for each of the specimen.

#### Tensile test

Prior to performing the tensile test, the as-cast composite cylindrical samples were prepared to dimension, 5 mm diameter and 30 mm gauge length using the lathe machine (Fig. 2c). The tensile experiment was done on a Universal testing machine (Model no: Instron 3369). A strain rate of 10^–3^/s was utilized to monotonically pull each clamped specimen until failure/fracture based on ASTM E8/E8M-16ae1 standard^32^. For data reliability, the test was carried out in triplicates for each composite designation. Yield strength (YS), ultimate tensile strength (UTS), and percentage elongation were the properties obtained through the test.

#### Fracture toughness

For the evaluation of the fracture toughness of the produced AMCs, circumferential notch tensile (CNT) samples were prepared in tandem with the method of Alaneme et al^33^. The samples were machined to dimension: 30 mm gauge length, 4 mm gauge diameter, 3.1 mm notch diameter, and 60° notch angle. Each specimen was subjected to a tensile loading strain rate of 10^–3^/s until fracture with the aid of an Instron 3369 universal testing machine. The fracture toughness was evaluated using Eq. (4) by Dieter^34^. Equation 5 was then used to validate the fracture toughness values^35^.

4 $${K}_{1C}=\frac{{P}_{f}}{{D}^\frac{3}{2}}\left[1.72\left(\frac{D}{d}\right)-1.27\right]$$

5 $$D\ge {\left(\frac{{K}_{1c}}{{\sigma }_{y}}\right)}^{2}$$

where ${P}_{f}$ is the fracture load (kN), $D$ is the gauge diameter (mm), and $d$ is the notch diameter (mm). The condition of $1.2\le \frac{D}{d}\le 2.1$ was reported to be valid. The test was done in triplicates to ensure repeatability and reproducibility of the generated results.

## Results and discussion

### X-ray diffraction of the hybrid composites

**Figure 3:**
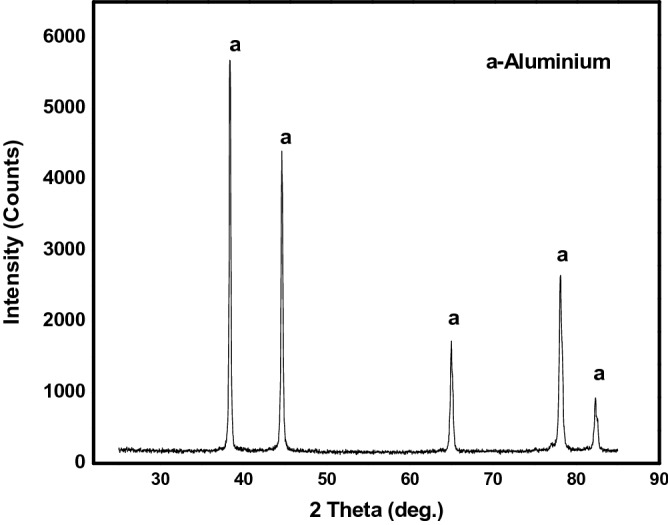
XRD of unreinforced Aluminium (Al6063).

**Figure 4:**
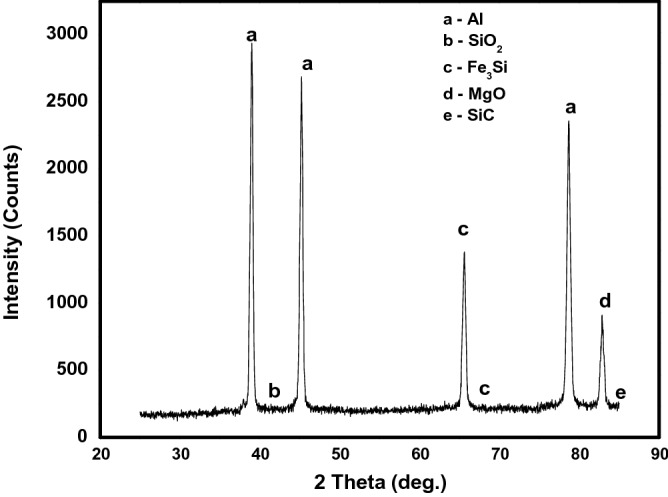
XRD of hybrid reinforced aluminium composite.

The various phases present in Al6063 and as formed composites were obtained and the XRD spectrum are presented in Figs. 3 and 4, respectively. In Fig. 3, only aluminium with a cubic crystal system can be identified with $a=b=c=4.0500 \mathrm{A}$ and $\propto =\beta =\gamma ={90}^{^\circ }$ with calculated density of 2.70 g/cm^3^. However, in the composite as displayed in Fig. 3, peaks depicting various phases present are identified as Al, Fe_3_Si (an intermetallic cubic crystal system), MgO, SiO_2_, and SiC. The presence of SiC, SiO_2_, and Fe_3_Si in the composites is strongly related to the presence of silica and Fe_2_O_3_ in the PKSA (Table 1). The formation of Fe_3_Si intermetallic phase emanated from the interaction and reaction of silica and Fe_2_O_3_. These same oxides were earlier identified in the characterized PKSA and described as hard particulates, which strengthen metallic materials^26^. In the same light, the diffractograms of AMCs produced with breadfruit seed hull particulates showed the presence of Fe_3_Si, Al_6_Fe, and $\propto$-Al indicating the presence of the reinforcement particulates in the composites^36^. The interaction of silica and MgO resulted in the formation of Mg_2_SiO_4_ as described in the study of Aigbodion & Ezema^8^. However, further report by Aigbodion & Ezema^8^, indicated that Al_4_C_3_ (a brittle reaction product) was not formed. This was not also seen in the composites produced in this present study. The presence of SiO_2_ and absence of carbon due to the calcination of PKS in producing PKSA may attribute to the non-formation of Al_4_C_3_ in the composites developed.in the current study. The presence of Al_4_C_3_ in MMCs is detrimental to the corrosion and mechanical behaviours of the MMCs^15^.

### Microstructural characterization of the composites

**Figure 5:**
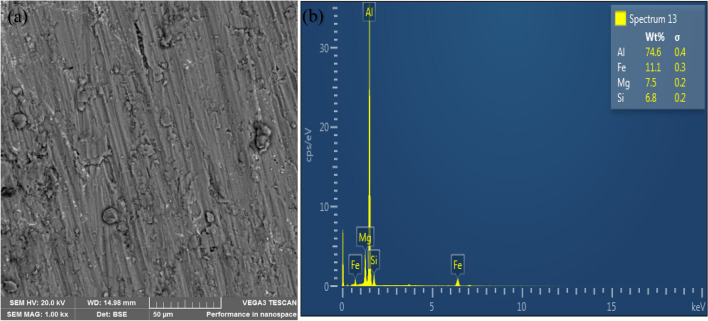
(**a**) Scanning electron micrograph and (**b**) EDS spectrum of Al6063.

**Figure 6:**
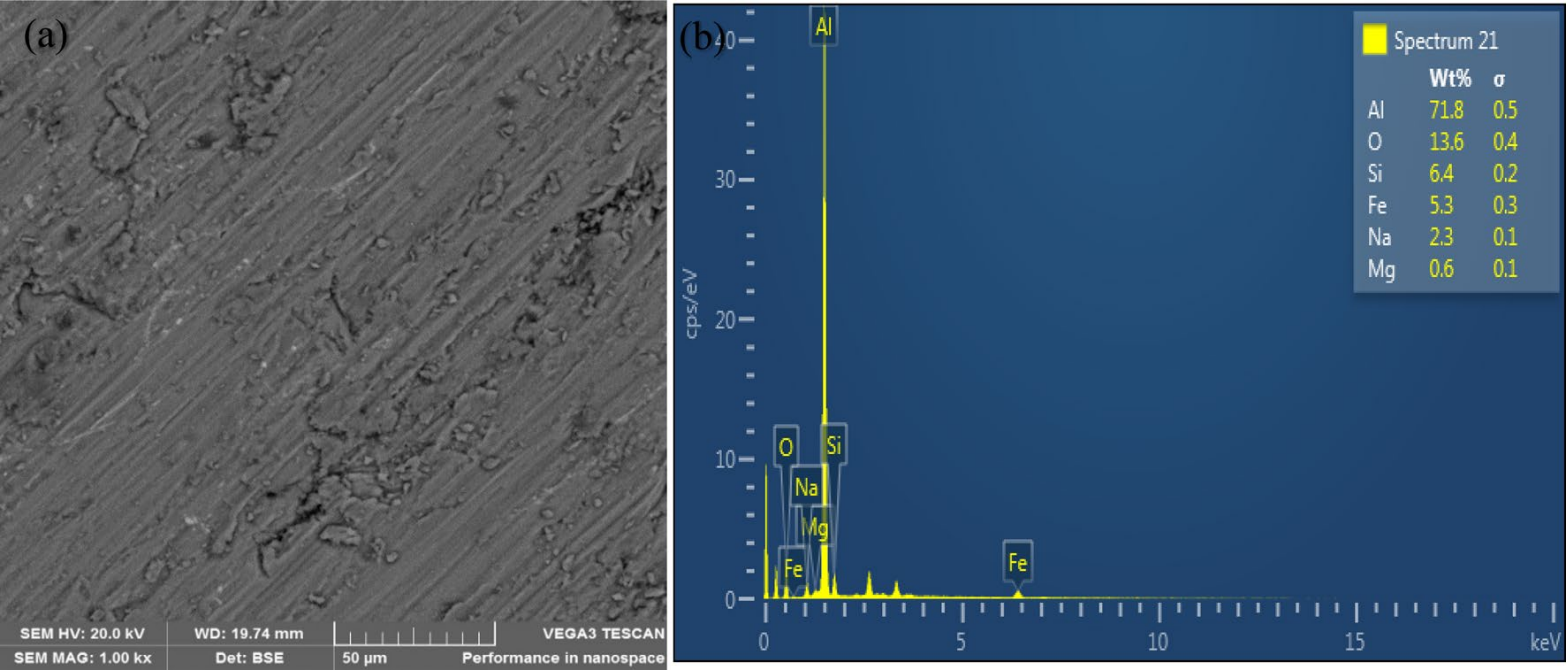
Representative morphology of reinforced composite (A1) (**a**, **b**).

**Figure 7:**
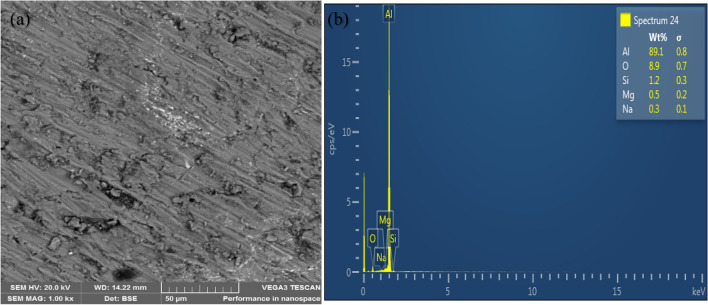
Representative morphology of reinforced composite (A5) (**a**, **b**).

The microstructure of the unreinforced alloy reveals structures without voids as shown in Fig. 5(a). Figures 6(a) and 7(a) show the representative microstructure of the monolithic reinforced and hybrid reinforced composites, respectively. The reinforcements (SiC and PKSA) present were uniformly dispersed in the matrix. There was no indication of crack formation and enlarged pores in the castings. This may be due to the appropriate process parameters utilized for synthesizing of the composite. This indicates better wetting condition between the matrix and the reinforcements. This assertion is corroborated by the use of Al/Zn matrix alloy with hybrid reinforcement of fly ash and SiC^37^; Al–Mg–Si matrix alloy with hybrid reinforcements of alumina, RHA, and graphite^38^; and Al–Cu–Mg matrix alloy with bean pod ash as reinforcement^7^ and many others; maintained no crack formation due to proper process parameters employed. Figures 6(a) and 7(a) further buttress the observations made in the low values of porosity in the as-formed composites. The evenly dispersed particulates observed in the SEM images further confirm that the double stir casting was reliable route in breaking the surface tension between the matrix alloy and the reinforcement particulates^39,40^. The double stir casting process also helped in reducing porosity by allowing air bubbles trapped in the slurry to escape during processing.

The elemental mappings from the EDS profile for both the unreinforced (Fig. 5b) and reinforced (Figs. 6b and 7b) alloys show peaks of aluminium (Al), silicon (Si), iron (Fe), magnesium (Mg), and oxygen (O). The profile confirmed that the major elements (Al, Mg, and Si) in the matrix alloy (Al6063) have the tendency of forming oxides of Al (Al_2_O_3_), Si (SiO_2_), Fe (Fe_2_O_3_), and Mg (MgO). These were the constituents of the PKSA used in synthesizing the composite. Oxide formation accounts for the oxygen peaks observed in the EDS spectra of the as-formed composites, which is conspicuously absent in the EDS for Al6063.

### Impact of PKSA on the physical properties of the composite

**Figure 8:**
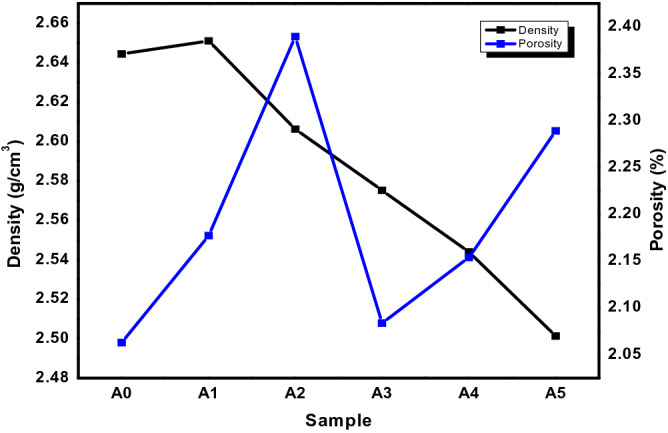
Density and porosity of the produced AMCs with PKSA variation.

The theoretical density of the composite samples is displayed in Table 3. Figure 8 shows the density and percentage porosity of the produced AMCs. The Al-matrix had a density of 2.6443 g/cm^3^, which was 5.4% higher than the composite in sample A5 with PKSA and SiC ratio (8:2) having a density of 2.5014 g/cm^3^. It was observed that as the PKSA particulates increased in the AMCs, the density of the AMCs reduced. However, sample A1 showed an increment in the density when compared to the unreinforced alloy and the reinforced alloy with the inclusion of PKSA and SiC. This could be attributed to the density of SiC that is higher than for the base alloy. The lower and reducing densities of other reinforced samples (A2—A5) may be attributed to the inclusion of the PKSA particulates. Thus, PKSA particulates lower the density of the composites. In a similar study by Prasad et al^30^, there was decrease in density when RHA and SiC were used as hybrid reinforcements. The drop in density was attributed to the inclusion of low density RHA particulates. Edoziuno et al.^18^ also reported similar observation when novel PKS reinforcement was solely used in the production of AMCs. The low density of the PKS resulted in low density AMCs. However, the usage of higher density reinforcement particulates in matrix alloy is said to improve the density of composite alloy^39,41^. Kumar et al.^41^ utilized Si_3_N_4_ as reinforcement in Al6063 alloy in the production of metal composites and observed that the higher the contents of the reinforcement, the higher the density, which ultimately enhanced the hardness of the produced composites.

**Table 3:** Theoretical density of the fabricated samples.

Sample	Theoretical density (g/cm^3^)
A0	2.7000
A1	2.7102
A2	2.6726
A3	2.6350
A4	2.5974
A5	2.5600

The percentage porosities of the produced AMCs were observed to be less than 2.5%, which were within the acceptable limits for cast MMCs. Alaneme & Sanusi^38^ reported the maximum permissible percentage porosity to be less than 4% in cast MMCs. Hence, this further affirmed that double stir casting technique is reliable and efficient in breaking the surface tension between the Al matrix and the reinforcement particles. In addition, the double stir casting process helped in reducing porosity by allowing air bubbles entrapped in the slurry to escape during processing^39,40^. Based on the physical properties obtained, light-weight composites can be produced from the use of PKSA particles as reinforcement in MMCs.

### Influence of PKSA on the mechanical properties of the composite

#### Hardness

**Figure 9:**
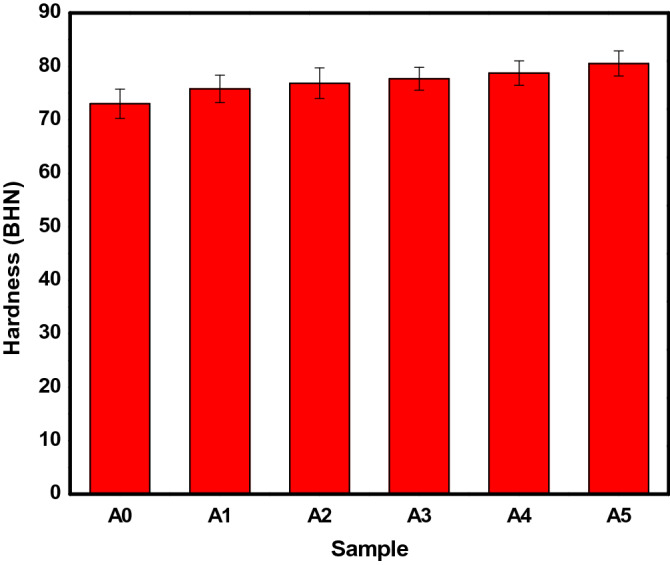
Hardness value for the produced AMCs.

The material strength, toughness, and resistance to wear could be influenced by the hardness value of the material. Figure 9 showed the hardness values of the produced AMCs. From Fig. 9, it can be observed that the hardness value of Al6063 alloy was enhanced as the percentage weight of the PKSA increased with a constant SiC value of 2 wt.%. The presence of the hard phases of silica and other strengtheners in the PKSA could be said to be the reason for the increase in hardness. According to Reddy et al^42^, the improvement of hardness of MMCs have been attributed to the incorporation of reinforcement particulates into the base alloy. Same observation was accredited to the higher hardness of ceramic particles present in the BPA reinforced composites against the observation in the unreinforced matrix alloy^7^. Although, silica and other oxides (such as Al_2_O_3_, MgO, and so on) present in the PKSA has lower density compared to SiC; however, the 2 wt.% SiC present in the hybrid reinforcement particulates increased the hardness value of the MMCs. More so, the hardness can be ascribed to the hard and brittle phases of the PKSA, which is obtainable from the SiO_2_, Fe_2_O_3,_ and MgO constituents (Table 1). The presence of the Fe_3_Si intermetallic could also improve the hardness of the composites. This could also be attributed to the increment in reinforcements surface area and matrix grain size reduction as a result of reinforcement particulate addition^43^. The increase in hardness could be attributed to the increase in the volume of precipitated phases or a high dislocation density. More plastic deformation resistances are obtained by the inclusion of these particulates leading to rise in hardness value. From this study, a 10.31% increment in hardness value was obtained for sample A5. This implied that the introduction of PKSA as substitute to SiC does not adversely affect the resistance to indentation of the composite. This is in agreement with the study of Alaneme et al^44^. The hardness value obtained in this study was better compared with that obtained in the study of Aigbodion & Ezema^8^. This is because of the presence of SiC particulate incorporated as the second reinforcement in this study. The hardness increased with decrease in the average size of aluminium composites when bamboo leaf ash was incorporated into Al-4.5Cu matrix in the study of Kumar and Birru^45^. This observation was seen in the study of Bannaravuri and Birru^46^, where the hardness increased as the reinforcement contents increased. The findings in this present study makes the MMCs produced suitable to be used in light-weight applications where mild hardness is required such as in architectural works, and window and door frames.

#### Yield and ultimate tensile strengths

**Figure 10:**
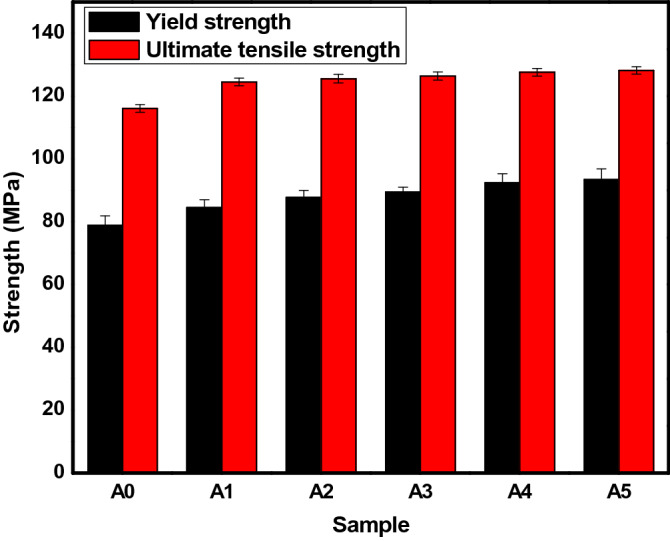
YS and UTS of the composite produced.

The YS and UTS of the hybrid-reinforced composites derived from the stress–strain curves generated by the universal testing machine, are shown in Fig. 10. It can be observed that the YS and UTS of sample A1 are higher than that of the un-reinforced sample (Sample A0). Sample A1 is a sample with 2 wt.% SiC inclusion into the base alloy. Improvement in the values of the YS and UTS may be ascribed to the hard nature and strength of the SiC particulates present. The influence of the PKSA particulates addition between 2 and 8 wt.% with the constant 2 wt.% SiC showed increment in the YS and UTS for the samples A2 – A5. The more the PKSA content, the better the strengths of the synthesized composites. These may be assigned to the presence of the hard nature of the brittle PKSA particulates as well as the presence of the SiC particulates, which were agglomerated into the Al6063 alloy. The hard nature of PKSA particulates could be as a result of the existence of hardening and strengthening oxides such as SiO_2_, Al_2_O_3_, Fe_2_O_3_, MgO, and so on^26^. Hence, the rise in tensile strength is proportional to the rise in hardness value. This observation is in accordance with the study of several researchers^37,39,41,42^. Based on this study, the rise in the yield strength and ultimate tensile strength of the composites could be linked to three mechanisms. These include the grain boundary strengthening known as the Hall–Petch effect, the coefficient of thermal expansion (CTE) as well as the Orowan strengthening mechanisms^47^. According to Atuanya et al^36^, various factors significantly affect the mechanical properties of metallic materials. These factors include grain boundaries, solid solutions, sub-structures, second phases, and many more. However, the information from the morphology of the composites developed reveal that the grain refinement of the reinforcement and the matrix are the core contributors to the tensile strength improvement. The grain boundaries serve as obstacles to the dislocation movement. Around the dislocation density, there is rise in grain boundaries which could result in increasing the strength of the composite^47^. The particulates of the PKSA reinforcement play significant function on the load transfer in the strengthening of the composites developed. There is an atomical bonding interface between the matrix and the PKSA reinforcement to produce grain-boundaries. The increase in the tensile strength of the developed composites as PKSA content increases may be attributed to the grain refinement of the aluminium matrix alloy. More loads are transferred when the volume fractions of the reinforcements are increased in the matrix alloy; resulting in higher strength. This is in agreement with the study of Atuanya et al.^36^. The more the PKSA particulates in the matrix, the smaller is the grain of the Al matrix^8,36^. More so, when there is a thermal matrix-reinforcement interface mismatch owing to the disparity between the thermal expansion coefficients of the matrix and the reinforcement, the composite material is strengthened. The particulates are hard and brittle, while the matrix is soft and ductile; however, the thermal mismatch of the CTE could result into elastic and plastic incompatibility between the matrix and the reinforcement^36^. The mismatch occurs during the cooling or solidification process of the composite production; hence, producing plastic deformation in the interface of the matrix^8,15^. In addition, the grain size of the matrix is bigger with a low volume fraction of particulates, which may lead to the Orowan bypass mechanism in operation within the large grains of the matrix. The higher reinforcing particulate fraction in the matrix, the grains get reduced to induce grain refinement^36^. This could also lead to higher yield and tensile strength values through load bearing transferred directly to the interface. With the addition of bamboo leaf ash and breadfruit seed hull ash into Al matrix, increase in YS and UTS were observed, respectively^36,46^.

#### Percentage elongation

**Figure 11:**
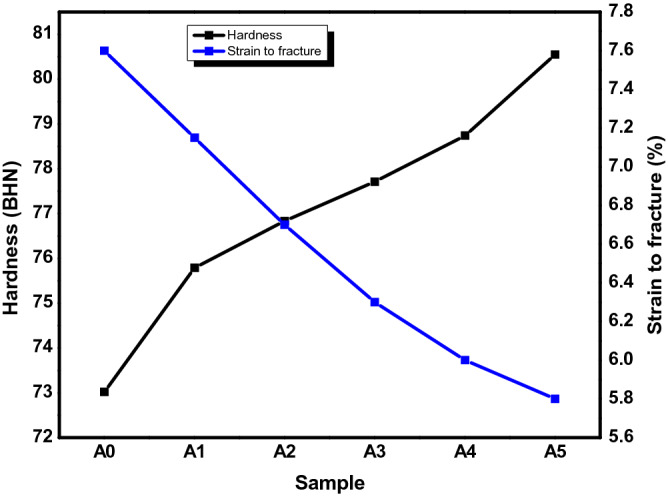
Percentage elongation of the produced AMCs.

The percentage elongation (strain to fracture) of the produced composites is presented in Fig. 11. The % elongation of the samples produced were observed to be between 5.8 and 7.6%. In addition, reduction in % elongation can be observed as the PKSA particulates in the hybrid reinforcement increased. The reduction in %elongation may be attributed to the reduction in the plastic strain sustenance ability of the composites due to the presence of hard constituents in the PKSA (Table 1) and SiC particulates. This could cause a reduction in the ductile phase of the matrix leading to decline in percentage of elongation. It can also be observed that there is a consistent reduction in the strain to fracture value, which may be ascribed to the flowability resistance of the matrix alloy with the inclusion of reinforcement particles as well as the reduction of the ductile nature of the aluminium matrix content^6^. The PKSA inclusion has slight detrimental effect on the ductility of the AMCs produced. Good ductility was observed when 1 wt.% corn cob ash (CCA) was utilized along with SiC in the development of AMCs when compared with unreinforced matrix^17^. However, as the CCA content increased, the ductility of the composites declined. Furthermore, there was decrease in ductility as the reinforcement (Si_3_N_4_) particulates increased in the study of Kumar et al.^41^. The inclusion of PKSA as reinforcement in the development of AMCs caused an inverse response in the hardness value and the percentage elongation. As the PKSA content increased, the hardness value increased while the % elongation reduced. The elastic property and the ability of the drawn of the matrix alloy reduces with the reinforcement content (PKSA and SiC) increase because PKSA and SiC particulates are brittle in nature. The results in this study is similar to the work of Atuanya et al.^36^, in which the increment in breadfruit seed hull ash slightly lower the percentage elongation. This is a consistent trend in the study. However, an inconsistent trend of %elongation was seen when hybrid reinforcements of groundnut shell ash and SiC was used to develop MMCs. Some of the samples show slightly improved ductility, while others revealed otherwise^14^. Similar pattern of reduction in the %elongation was reported for alumina and quarry dust^44^, bamboo leaf ash^46^ and breadfruit seed hull ash^36^ when used as hybrid reinforcements in Al matrix. The reduction in the percentage elongation may be due to the increase in hardness and strength of the composite. The results obtained in this study is in tandem with these studies and are thus recommended for light weighted application.

### Fracture toughness

**Figure 12:**
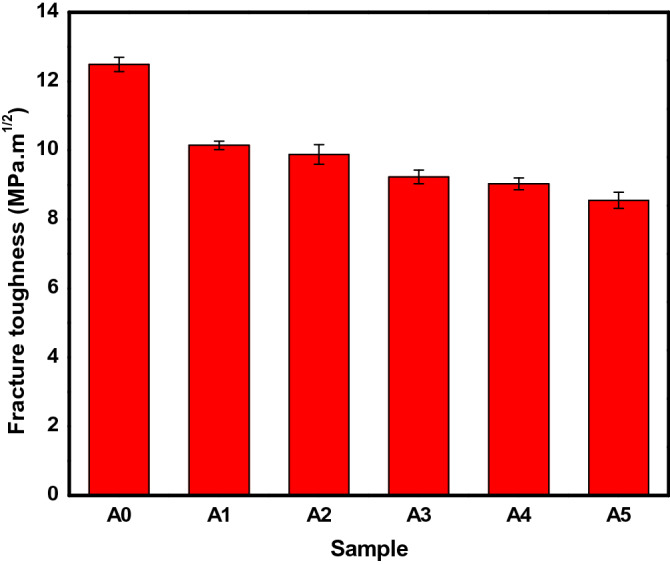
Fracture toughness of the produced AMCs.

Fracture toughness is the ability of composite material to resist crack propagation. The fracture toughness values of the samples produced are shown in Fig. 12. The range of the fracture toughness values can be observed to be between 8.55 and 12.49 MPa m^1/2^, which declined as the PKSA content in the hybrid reinforcement increased. Alaneme et al.^39^ reported similar observation, where fracture toughness reduced with increase in alumina particulate inclusion. In this study, the unreinforced matrix gave the highest fracture toughness of 12.49 MPa m^1/2^. The lower fracture toughness of the composites may be due to the presence of brittle PKSA particulates in the composites. Brittle materials such as PKSA have low resistance to crack propagation. Thus, low fracture toughness corresponds to low ductility in brittle materials unlike ductile materials that show better opposition to crack propagation. Ductile materials have the ability of redistributing applied stresses and strains by plastic deformation. In ceramic reinforced MMCs, the basic micro-mechanism was reported to be due to particle cracking, interfacial cracking, and particle bonding. The increased crack nucleation due to the increase in the brittle PKSA reinforcement particulates may be responsible for the trend observed in this study. This assertion has been reported by Alaneme & Bodunrin^48^ and Alaneme et al^39^ . The fracture toughness of sample A1, which is higher than for other samples (A2–A5) is indicative of the ductile nature of SiC particulates, which may result in a mixed fracture modes^39^.

### SEM characterization of the fractured surface

**Figure 13:**
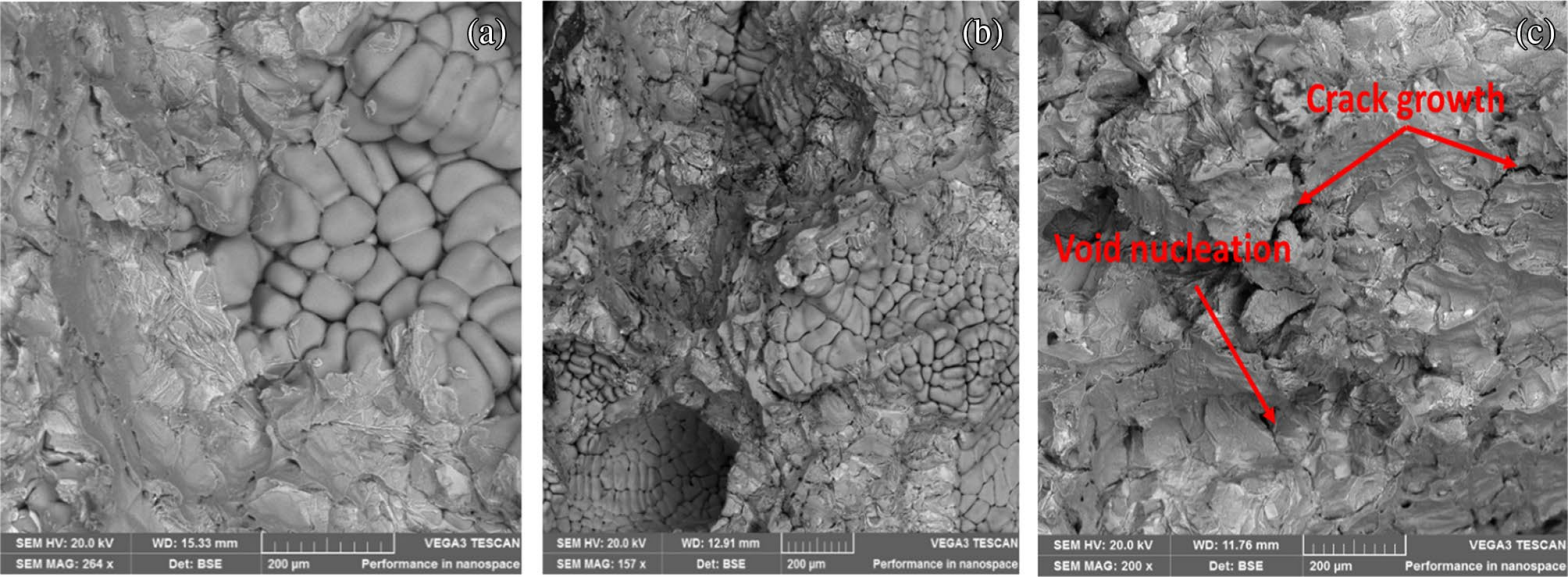
Fractured surface micrograph for (**a**) Unreinforced matrix, (**b**) reinforced composite (A1), (**c**) reinforced composite (A5).

For the tensile fracture, the surface morphologies of the representative samples are displayed in Fig. 13(a–c). Figure 13(a) shows the SEM micrograph of the fractured surface for Al 6063 alloy, while Fig. 13(b,c) show the SEM micrographs of fracture surfaces of the composites. Cup and cap surfaces can be observed on the fractured surfaces of the composites. The fractured surface of the Al matrix shows large population of well-developed dimples. A mixed fracture mechanism was seen on the fractured surface of the composite samples. This is as a result of the ductile tearing of the matrix alloy and the brittle fracture of the reinforcement particles. There is no noticeable necking formation for the composites produced. This could be attributed to the rapid occurrence of pore nucleation, growth and coalescence. According to Yigezu et al^3^, pores nucleation, growth, and coalescence majorly contribute to the final fracture of the matrix. More so, the presence of weak and detrimental phases from the PKSA and intermetallic phases activate a different fracture mechanism, which cause the composite to fail via the nucleation, growth, and coalescence of the pores. The growth of the pores is by plastic straining through the local necking of the intervoid matrix, leading to final fracture. There is strong interfacial bonding among the PKSA, SiC and the Al6063 matrix alloy as particle–matrix interfacial bonding was not observed^36^. Although, the samples showed good hardness and strength as the PKSA particulates increased; the PKSA particulates lowered the ductility of the hybrid reinforced composites.

## Conclusion

The study was on the physico-mechanical properties and morphological examination of aluminium hybrid composites produced using Al 6063 as the matrix alloy with SiC and PKSA as reinforcements of different proportions. The occurrence of some compounds such as SiO_2_, MgO, SiC, and intermetallic (Fe_3_Si) were seen in the composites based on the XRD analysis. These compounds serve as strengtheners and hardeners for the composites. There was homogenous dispersion of both reinforcements in the alloy. It can be concluded that the density of the AMCs produced reduced as the PKSA particulates contents increased in the matrix. The level of the porosity of composites produced through double stir casting route was found to be within the permissible limit (4%) for MMC castings. The increased addition of PKSA when the SiC was constant at 2% into the matrix improved the hardness value, yield strength, ultimate tensile strength, and reduced the percentage elongation and fracture toughness of the synthesized composites. A fracture mechanism was activated due to the presence of weak phases from the PKSA and intermetallic phases, which resulted in composite failure through nucleation, growth, and coalescence of pores. The AMCs produced will be very useful in light-weight engineering applications such as aluminium frames and roofing sheets production.

## Data Availability

The data will be made available upon request.

## References

[1] Ikubanni PP, Oki M, Adeleke AA (2020). A review of ceramic/bio-based hybrid reinforced aluminium matrix composites. Cogent Eng..

[2] Prasad MGA, Bandekar N (2015). Study of microstructure and mechanical behavior of aluminum/garnet/carbon hybrid metal matrix composites (HMMCs) fabricated by chill casting method. J. Mater. Sci. Chem. Eng..

[3] Yigezu SB, Mahapatra MM, Jha PK (2013). Influence of reinforcement type on microstructure, hardness, and tensile properties of an aluminum alloy metal matrix composite. J. Miner. Mater. Charact. Eng..

[4] Madeva Nagaral V, Auradi SA, Kori VH (2019). Investigations on mechanical and wear behavior of nano Al_2_O_3_ particulates reinforced AA7475 alloy composites. J. Mech. Eng. Sci..

[5] Orhadahwe TA, Ajide OO, Adeleke AA, Ikubanni PP (2020). A review on primary synthesis and secondary treatment of aluminium matrix composites. Arab J. Basic Appl. Sci..

[6] Singh G, Goyal S (2018). Microstructure and mechanical behavior of AA6082-T6/SiC/B_4_C-based aluminum hybrid composites. Particulate Sci. Technol..

[7] Aigbodion VS (2019). Bean pod ash nanoparticles a promising reinforcement for aluminium matrix biocomposites. J. Mater. Res. Technol..

[8] Aigbodion VS, Ezema IC (2020). Multifunctional A356 alloy/PKSAnp composites: Microstructure and mechanical properties. Defence Technol..

[9] Mavhungu ST, Akinlabi ET, Onitiri MA, Varachia FM (2017). Aluminum matrix composites for industrial use: Advances and trends. Procedia Manufact..

[10] Rajesh, A.M. & Kaleemulla, M. Experimental investigations on mechanical behavior of aluminium metal matrix composites. *IOP Conf. Series: Mater. Sci. Eng.*, **149**, 1–12 (2016).

[11] Parswajinan C, Ramnath BV, Abishek S, Niharishsagar B, Sridhar G (2018). Hardness and impact behaviour of aluminium metal matrix composite Hardness and impact behaviour of aluminium metal matrix composite. IOP Conf. Ser. Mater. Sci. Eng..

[12] Poornesh M, Harish N, Aithal K (2016). Study of mechanical properties of aluminium alloy composites. Am. J. Mater. Sci..

[13] Akinlabi, E.T., Fono-Tamo, R.S. & Tien-Chien, J. Microstructural and dry sliding friction studies of aluminum matrix composites reinforced PKS ash developed via friction stir processing. In C. Chesonis (Ed.), *The Minerals, Metals *&* Materials Series* (Light Metals), 401–406 (2019).

[14] Alaneme KK, Bodunrin MO, Awe AA (2018). Microstructure, mechanical and fracture properties of groundnut shell ash and silicon carbide dispersion strengthened aluminium matrix composites. J. King Saud Univ. Eng. Sci..

[15] Bodunrin MO, Alaneme KK, Chown LH (2015). Aluminium matrix hybrid composites: A review of reinforcement philosophies; mechanical, corrosion and tribological characteristics. J. Mater. Res. Technol..

[16] Dinaharan I, Kalaiselvan K, Murugan N (2017). Influence of rice husk ash particles on microstructure and tensile behavior of AA6061 aluminum matrix composites produced using friction stir processing. Composites Communicat..

[17] Fatile OB, Akinruli JI, Amori AA (2014). Microstructure and mechanical behaviour of stir-cast Al–Mg–Si alloy matrix hybrid composite reinforced with corn cob ash and silicon carbide. Int. J. Eng. Technol. Innov..

[18] Edoziuno FO, Adediran AA, Odoni BU, Utu OG, Olayanju A (2021). Physico-chemical and morphological evaluation of palm kernel shell particulate reinforced aluminium matrix composites. Materials Today: Proceedings.

[19] Adebayo O (2012). Asessment of palm kernel shells as aggregate in concrete and laterite blocks. J. Eng. Stud. Res..

[20] Olutoge FA, Quadri HA, Olafusi OS (2012). Investigation of the strength properties of palm kernel shell ash concrete. Eng. Technol. Appl. Sci. Res..

[21] Oti JE, Kinuthia JM, Robinson R, Davies P (2015). The use of palm kernel shell and ash for concrete production. Int. Sci. Index Civ. Environ. Eng..

[22] Fono-Tamo RS, Idowu OO, Koya FO (2014). Development of pulverized palm kernel shells based particleboard. Int. J. Mater. Mech. Eng..

[23] Obi OF (2015). Evaluation of the physical properties of composite briquette of sawdust and palm kernel shell. Biomass Conver. Bioref..

[24] Hardjasaputra, H., Fernando, I., Indrajaya, J., Cornelia, M. & Rachmansyah, R. The effect of using palm kernel shell ash and rice husk ash on geopolymer concrete. *MATEC Web Conferences*, **251**, 1–16 (2018).

[25] Misnon II, Zain NKM, Jose R (2019). Conversion of oil palm kernel shell biomass to activated carbon for supercapacitor electrode application. Waste Biomass Valoriz..

[26] Ikubanni PP, Oki M, Adeleke AA, Adediran AA, Adesina OS (2020). Influence of temperature on the chemical compositions and microstructural changes of ash formed from palm kernel shell. Results Eng..

[27] Imoisili PE, Ukoba KO, Jen T (2020). Synthesis and characterization of amorphous mesoporous silica from palm kernel shell ash. Boletín de La Sociedad Española de Cerámica y Vidrio.

[28] Mortimer R, Saj S, David C (2018). Supporting and regulating ecosystem services in cacao agroforestry systems. Agrofores. Sys..

[29] Oladele IO, Okoro AM (2016). The effect of palm kernel shell ash on the mechanical properties of as-cast aluminium alloy matrix composites. Leonardo J. Sci..

[30] Prasad DV, Shoba C, Ramanaiah N (2014). Investigations of mechanical properties of aluminum hybrid composites. J. Market. Res..

[31] ASTM E10-18, *Standard Test Methods for Brinell Hardness of Metallic Materials*. ASTM International (2018). www.astm.org

[32] ASTME8/E8M-16ae1, *Standard Test Methods for Tension Testing of Metallic Materials*. ASTM International (2016). www.astm.org

[33] Alaneme KK, Oganbule CA, Adewale A (2020). Circumferential notch test based fracture toughness investigation of Al–Mg–Si alloy composites reinforced with alumina and quarry dust. J. Chem. Technol. Metallur..

[34] Dieter GE (1988). *Mechanical metallurgy* (SI Metric).

[35] Nath SK, Das UK (2006). Effect of microstructure and notches on the fracture toughness of medium carbon steel. J. Naval Architect. Marine Eng..

[36] Atuanya CU, Ibhadode AOA, Dagwa IM (2012). Effects of breadfruit seed hull ash on the microstructures and properties of Al–Si–Fe alloy/breadfruit seed hull ash particulate composites. Results Phys..

[37] Kanth UR, Rao PS, Krishna MG (2019). Mechanical behaviour of fly ash/SiC particles reinforced Al–Zn alloy-based metal matrix composites fabricated by stir casting method. J. Mat. Res. Technol..

[38] Alaneme KK, Sanusi OK (2015). Microstructural characteristics, mechanical and wear behaviour of aluminium matrix hybrid composites reinforced with alumina, rice husk ash and graphite. Eng. Sci. Technol. Int. J..

[39] Alaneme KK, Fajemisin AV, Maledi NB (2019). Development of aluminium-based composites reinforced with steel and graphite particles: Structural, mechanical and wear characterization. J. Mat. Res. Technol..

[40] Alaneme, K. K. & Aluko, A. O. Fracture toughness (K1C) and tensile properties of as-cast and age-hardened aluminium (6063)-silicon carbide particulate composites. *Scientia Iranica A* **19**, 992–996 (2012).

[41] Kumar GBV, Prasad P, Suresh N, Pramod R, Rao CSP (2019). Assessment of mechanical and tribological characteristics of Silicon Nitride reinforced aluminum metal matrix composites. Compos. Part B.

[42] Reddy PV, Prasad PR, Krishnudu DM, Goud EV (2019). An investigation on mechanical and wear characteristics of Al 6063/TiC metal matrix composites using RSM. J. Bio- Tribo-Corros..

[43] Sarada, B.N., Murthy, P.L.S. & Ugrasen, G. Hardness and wear characteristics of hybrid aluminium metal matrix composites produced by stir casting technique. *Mater. Today Proc.* **2**, 2878–2885 (2015).

[44] Alaneme KK, Adegun MH, Archibong AG, Okotete EA (2019). Mechanical and wear behaviour of aluminium hybrid composites reinforced with varied aggregates of alumina and quarry dust. J. Chem. Technol. Metallur..

[45] Kumar BP, Birru AK (2017). Microstructure and mechanical properties of aluminium metal matrix composites with addition of bamboo leaf ash by stir casting method. Trans. Nonferrous Met. Soc. China.

[46] Bannaravuri PK, Birru AK (2018). Strengthening of mechanical and tribological properties of Al-4.5%Cu matrix alloy with the addition of bamboo leaf ash. Results Phys..

[47] Anestiev, L., Lazarova, R., Petrov, P., Dyakova, V., & Stanev, L. On the strengthening and the strength reducing mechanisms at aluminium matrix composites reinforced with nano-sized TiCN particulates. *Philos. Mag.* **101**, 129–153 (2021).

[48] Alaneme KK, Bodunrin MO (2013). Mechanical behaviour of alumina reinforced AA 6063 metal matrix composites developed by two-stir casting process. Acta Tech. Corvininesis Bull. Eng..

